# Trends of Chronic Liver Disease in a Tertiary Care Centre in the Eastern Part of India: A Retrospective Study

**DOI:** 10.7759/cureus.36662

**Published:** 2023-03-25

**Authors:** Sameer K Mehta, Shashank Sunny, Zaid Nafe, Kunal Priyadarshi, Prashant K Singh

**Affiliations:** 1 Department of General Medicine, Tata Main Hospital, Jamshedpur, IND; 2 Department of Internal Medicine, Tata Main Hospital, Jamshedpur, IND; 3 Department of Community Medicine, Tata Main Hospital, Jamshedpur, IND; 4 Department of Community Medicine, Dr. Vaishampayan Memorial Government Medical College, Solapur, IND; 5 Department of Critical Care Medicine, Tata Main Hospital, Jamshedpur, IND

**Keywords:** child-pugh-turcotte, ascites, alcohol, portal hypertensive gastropathy, child-pugh scoring system, cirrhosis

## Abstract

Aim of the study: To assess the relationship between the severity of liver cirrhosis and its outcomes based on laboratory parameters, Child-Turcotte-Pugh (CTP) score, and upper gastrointestinal (UGI) endoscopy findings.

Background: Cirrhosis is the end stage of chronic liver disease (CLD) and is characterised by progressive liver fibrosis and distortion of the liver architecture. It is a major cause of morbidity and mortality all over the world. Cirrhosis is compensated in the initial stages and later progresses to the decompensated stage with various complications. The CTP scoring system predicts mortality in patients with cirrhosis.

Materials and methods: This retrospective study was done in the Department of Medicine and Gastroenterology of Tata Main Hospital (TMH), Jamshedpur, Jharkhand, India. It was conducted over a period of two years between 1 January 2019 and 31 December 2020, on 150 confirmed cases of cirrhosis.

Results: The most common age group was 41-60 years (86, 57.33%) and the mean age ± standard deviation (SD) for all patients was 49.82 ± 11.63 years. In a total of 150 CLD cases, males were 96 (64%). The most common cause of CLD was alcohol (76, 50.67%). Based on presenting symptoms, most CLD patients presented with generalized weakness (144, 96.00%). The most common signs were icterus (68, 45.33%) and ascites (44, 29.33%). Most patients belonged to CTP class A (77, 51.33%), followed by CTP class B (44, 29.33%) and class C (29, 19.34%). The most common UGI endoscopy finding was portal hypertensive gastropathy (mild or severe) (135, 75%). Total deaths were 24 (16.00%), with 17 deaths (70.83%) in patients belonging to CTP class C.

Conclusion: CLD is a common entity in eastern India with male preponderance and affects mostly people of the middle age group. Alcohol intake is a major cause of CLD, followed by non-alcoholic fatty liver disease and chronic hepatitis B and C. A significant rise in morbidity and mortality due to alcoholic liver disease (ALD) was observed in the study and needs urgent social and medical intervention. The incidence of ALD in our study was 50.67%.

## Introduction

Cirrhosis is one of the most frequent causes of death due to hepatic diseases worldwide. Approximately two million deaths occur per year due to liver diseases, one million due to complications of cirrhosis and one million due to viral hepatitis and hepatocellular carcinoma [1]. Cirrhosis is currently the 11th most common cause of death globally [1]. The structural integrity of the liver is disrupted in cirrhosis and there is progressive replacement of the liver parenchyma by the fibrous tissue. Cirrhosis can be compensated or decompensated. The majority of patients are asymptomatic in the compensated stage and diagnosis at this stage is typically picked up during routine medical visits for other conditions. The mortality and morbidity caused by cirrhosis dramatically rise after decompensation takes place, and the one-year case-fatality rate might reach as high as 80% depending on the cause of decompensation. Lastly, there are only two outcomes for patients, i.e., death or cure by liver transplantation, which impose a heavy financial burden on individuals, healthcare systems, and health spending and governance [1].

Globally, 1.6 billion people had chronic liver disease (CLD) in 2017, with non-alcoholic fatty liver disease (NAFLD) (60%), hepatitis B virus (HBV) (29%), hepatitis C virus (HCV) (9%), and alcoholic liver disease (ALD) (2%) being the most frequent causes. Additionally, cirrhosis contributed to more than 132 million (95% UI: 127-145) deaths worldwide in 2017, with 883,000 (838,000-967,000, 66.7%) deaths among men and 440,000 (416,000-518,000, 33%) deaths among women. This is a significant increase as compared to 1990 when the total deaths from CLD in both sexes were 899,000 (829,000-948,000). In 2017, these fatalities made up 2.4% (2.3-2.6) of all deaths worldwide, up from 1.9% (1.8-2.0) in 1990. Cirrhosis is thought to affect between 16.5 and 23.6 people per 100,000 in East Asia and Southeast Asia, respectively. According to statistics from the Global Burden of Disease survey, there were 20.7 cases of cirrhosis per 100,000 people in 2015, up 13% from the year 2000. Over the previous 20 years, the prevalence of cirrhosis has grown 1.5-2 times [2].

Recent research has demonstrated that systemic inflammatory response syndrome (SIRS), with or without a confirmed bacterial infection, is an independent predictor of survival in critically unwell cirrhotic patients and is also linked to the emergence of problems caused by portal hypertension. In individuals with cirrhosis who develop multiorgan failure, liver function does not appear to be the primary driver of prognosis. In patients with severe or advanced cirrhosis, conventional measures for detecting SIRS lack sensitivity and specificity because of hypersplenism, hyperventilation coupled with encephalopathy, hyperkinetic circulation, and the use of beta-blockers [3]. To predict mortality in CLD patients, the Child-Pugh scoring system, also called the Child-Turcotte-Pugh (CTP) score, was developed. To help in the identification of patients who would benefit from elective surgery for portal decompression, Child and Turcotte first proposed the idea in 1964 [4]. The CTP score is based on five factors, including ascites, hepatic encephalopathy, prothrombin time or international normalized ratio (INR), serum albumin, and serum bilirubin level, which are among the three laboratory criteria and two clinical criteria. The CTP scoring system divides patients into three categories: class A (five to six points), class B (seven to nine points), and class C (10-15 points) [5]. With the use of the CTP score and upper gastrointestinal (UGI) endoscopy results, our study attempted to identify patterns in the relationship between laboratory results and the severity of liver cirrhosis and their association with outcomes.

## Materials and methods

This retrospective study was done at the Department of Medicine and Gastroenterology of Tata Main Hospital (TMH), Jamshedpur, Jharkhand. It was done over 150 confirmed cases of CLD admitted to the Department of Medicine and Gastroenterology between 1 January 2019 and 31 December 2020. The cases were diagnosed by ultrasonogram (USG) of the whole abdomen, UGI endoscopy, clinical features, and laboratory investigations. USG findings of CLD included shrunken liver with a nodular surface, ascites, and splenomegaly. Laboratory investigations suggestive of CLD were reversed albumin-globulin ratio with low albumin levels and deranged INR. Their records were retrieved from the hospital management system (HMS), a database of the patients coming to the TMH. Inclusion criteria included cases of CLD (compensated or decompensated) above the age of 18 years. Exclusion criteria included the existence of any concurrent infectious or systematic inflammatory diseases, significant haematologic disorders, thyroid dysfunction, and/or severe renal insufficiency (end-stage renal disease or chronic dialysis treatment) and non-hepatic malignant tumours and patients on warfarin, steroids, and hormone replacement therapy.

Clinical findings, laboratory investigations, including complete blood counts, liver function tests, kidney function tests, serum electrolytes, coagulation parameters (prothrombin time and INR), blood sugar, autoimmune profile, hepatitis B surface antigen, anti-HCV antibody, HBV DNA quantitative, and HCV RNA quantitative (where required), USG of the abdomen, chest X-ray posteroanterior view, ascitic fluid, total leucocyte count (TLC), differential leucocyte count (DLC), cultures, serum ascites albumin gradient (SAAG), and UGI endoscopic data were all collected. The statistical analysis was completed using Statistical Package for Social Sciences (SPSS, IBM Corp., Armonk, NY) and Microsoft Excel (Microsoft Corporation, Redmond, WA). The Student's t-test and the chi-square test were employed in the data analysis to compare continuous and dichotomous variables, respectively. Descriptive statistics were also used as necessary. To investigate distributional differences, the data were tabulated and presented as frequency (n) and percentage (%), and the continuous parameters were presented as mean ± standard deviation (SD). The correlation of different CTP classes with outcomes was done with a one-way ANOVA method. Statistical significance was determined to be a p-value of 0.05 or less. Informed permission was acquired from participants before they were enrolled in our research.

## Results

In the present study, the most common age of patients with CLD was 41-60 years (86 patients, 57.33%), followed by 21-40 years (43 patients, 28.67%) and 61-80 years (11 patients, 07.33%). Only six (4%) patients were under 20 years of age. The mean age ± SD for all studied patients was 49.82 ± 11.63 years (Table 1).

**Table 1:** Age distribution of studied CLD patients. SD: standard deviation; CLD: chronic liver disease.

Age	Number of patients	Percentage
00-20	06	04.00%
21-40	43	28.67%
41-60	86	57.33%
61-80	11	07.33%
>80	04	02.67%
Mean age ± SD	49.82 ± 11.63 years	

**Figure 1:**
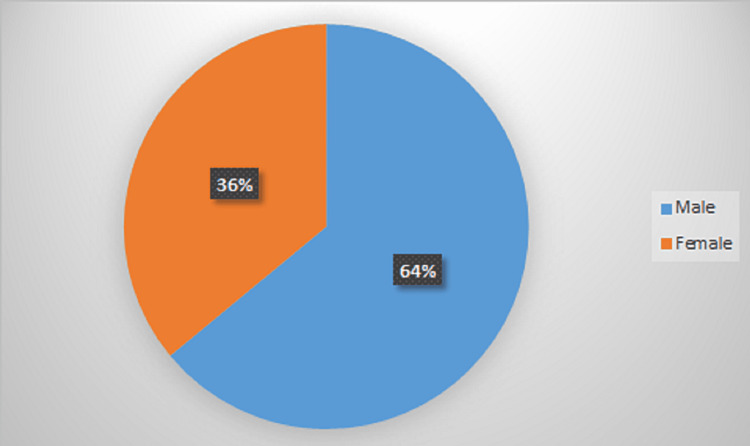
Gender distribution of studied CLD patients. CLD: chronic liver disease.

Out of the total 150 CLD cases, 96 (64%) were male and 54 (36%) were female (Figure 1).

As per the aetiology, the most common cause of CLD was alcohol (76, 50.67%), followed by NAFLD (37, 24.67%). Hepatitis B, hepatitis C, and cryptogenic factor-causing CLD cases were 11 (07.33%), seven (04.67%), and 15 (10.00%), respectively. The least number of cases were of autoimmune CLD (4, 02.66%) (Table 2).

**Table 2:** Distribution of 150 CLD patients on the basis of aetiology. CLD: chronic liver disease; ALD: alcoholic liver disease; NAFLD: non-alcoholic fatty liver disease; HEP-B: hepatitis B; HEP C: hepatitis C.

Aetiology	Number of patients	Percentage
ALD	76	50.67%
NAFLD	37	24.67%
HEP-B related	11	07.33%
HEP-C related	07	04.67%
Autoimmune	04	02.66%
Cryptogenic	15	10.00%

In the present study, the most common symptom in patients with CLD was generalized weakness (144, 96.00%), followed by jaundice (82, 54.67%) and blood in vomiting and/or black stool (61, 40.66%). Of the patients, 49 (32.67%) had abdominal distension due to ascites. Pedal oedema and altered sensorium also presented in 27 (18.00%) cases each. The least common symptom was fever (6, 4.00%) (Table 3). Many patients had multiple symptoms.

**Table 3:** Distribution of symptoms presented by CLD patients. CLD: chronic liver disease. * Symptoms were overlapping.

Spectrum of symptoms*	Number of patients	Percentage
Generalized weakness	144	96.00
Jaundice	82	54.67
Pedal oedema	27	18.00
Pain abdomen	11	07.33
Reduced urine output	15	10.00
Fever	06	04.00
Abdominal distension	49	32.67
Blood in vomiting and/or black stool	61	40.66
Altered sensorium	27	18.00

In the present study, the most common signs in patients with CLD were icterus (45.33%), ascites (29.33%), pedal oedema (13.33%), and asterixis (07.33%). There was an overlap of multiple clinical signs in many patients (Table 4).

**Table 4:** Clinical signs shown by selected CLD patients. CLD: chronic liver disease. * Clinical signs were overlapping sometimes.

Clinical signs*	Number of patients	Percentage
Icterus	68	45.33%
Oedema	20	13.33%
Clubbing	00	00.00%
Asterixis	11	07.33%
Ascites	44	29.33%

**Figure 2:**
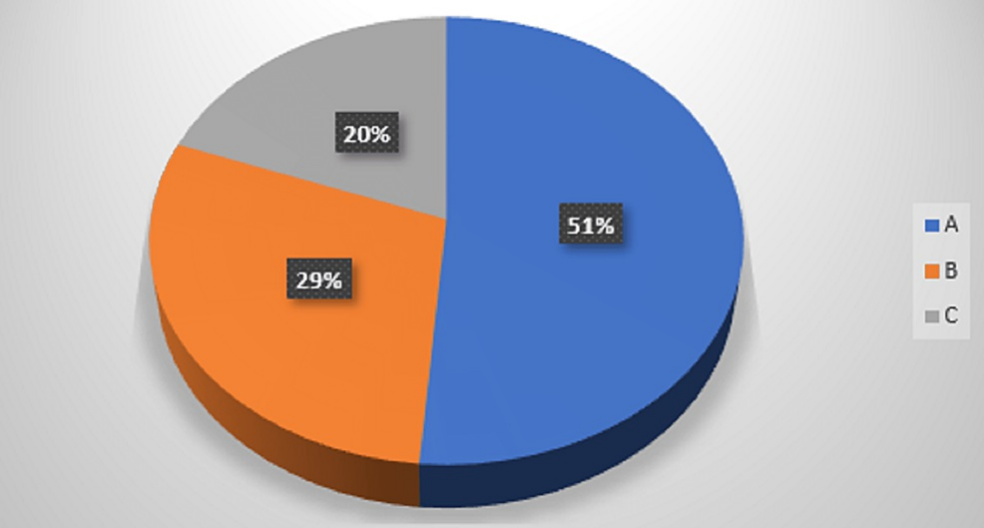
CTP classes (compensated or decompensated) in CLD patients. CTP: Child-Turcotte-Pugh; CLD: chronic liver disease.

In the present study, most patients belonged to CTP class A (77, 51.33%), followed by CTP class B (44, 29.33%) and class C (29, 19.34%) (Figure 2).

The most common UGI endoscopy findings in the present study were portal hypertensive gastropathy (mild or severe) (135, 75%) and grade I varices (63, 42%) (Table 5). Out of the 150 patients, 26 (17.33%) patients had grade II varices, 18 (12%) had grade III varices, and nine (6%) had grade IV varices. Of the patients, 37 (24.67%) underwent endoscopic variceal ligation (EVL). Antral gastritis or pangastritis was found in 12 (08.00%) patients.

**Table 5:** UGI endoscopy findings of studied CLD cases. UGI: upper gastrointestinal; CLD: chronic liver disease; GAVE: gastric antral vascular ectasia; PHG: portal hypertensive gastropathy; EVL: endoscopic variceal ligation. * UGI endoscopy findings were overlapped.

*UGI endoscopy findings	Number of patients	Percentage
Antral or pangastritis	12	08.00%
PHG	135	90.00%
GAVE	13	08.67%
Grade I varices	63	42.00%
Grade II varices	26	17.33%
Grade III varices	18	12.00%
Grade IV varices	09	06.00%
Portal hypertensive duodenopathy	17	11.33%
EVL done	37	24.67%

Means and SDs of serum bilirubin, serum albumin, alanine transaminase (ALT), aspartate aminotransferase (AST), haemoglobin, WBC, platelets, INR, serum creatinine, and serum electrolytes in different CTP classes were evaluated and it was found that all findings were statistically significant with p-value < 0.001, but serum creatinine and serum potassium levels showed statistically insignificant results (Table 6).

**Table 6:** Laboratory data in different classes of CTP. CTP: Child-Turcotte-Pugh; ALT: alanine transaminase; AST: aspartate aminotransferase; WBC: white blood cell; ml: millilitre; dl: decilitre; mEq: milliequivalents; S: significant; NS: non-significant.

Variables	CTP class A	CTP class B	CTP class C	P-value
Bilirubin in mg/dl	02.14 ± 00.87	02.67 ± 01.07	03.65 ± 01.57	<0.001 S
Albumin in gm/dl	03.07 ± 01.11	02.32 ± 01.01	01.92 ± 00.72	<0.001 S
ALT (IU/L)	41.24 ± 10.54	47.24 ± 09.82	44.11 ± 06.54	<0.001 S
AST (IU/L)	47.81 ± 07.63	58.47 ± 09.77	51.24 ± 10.24	<0.001 S
Haemoglobin	10.12 ± 02.12	09.14 ± 02.54	08.41 ± 02.55	<0.001 S
WBC (x10^3^ /ml)	07.55 ± 05.11	08.41 ± 04.21	08.74 ± 03.87	<0.001 S
Platelets (x10^3^ /ml)	102.50 ± 24.12	98.20 ± 31.24	78.24 ± 21.14	<0.001 S
International normalized ratio (INR)	01.41 ± 00.41	01.51 ± 00.54	01.87 ± 00.77	<0.001 S
Serum creatinine (mg/dl)	01.21 ± 00.21	01.31 ± 00.57	01.61 ± 00.65	0.524 NS
Serum sodium (mEq/l)	128.54 ± 03.21	126.41 ± 03.11	125.41 ± 02.41	<0.001 S
Serum potassium(mEq/l)	04.41 ± 01.11	04.25 ± 01.23	04.88 ± 01.57	0.624 NS

The total number of deaths in the present study was 24 (16.00%). A total of 17 (58.62%) deaths belonged to CTP class C and seven deaths (18.92%) belonged to CTP class B (Table 7). There were no deaths in CTP class A. The survival in CTP classes A, B, and C was 100%, 81.08%, and 41.38%, respectively.

**Table 7:** The correlation of different CTP classes with outcomes. CTP: Child-Turcotte-Pugh.

Outcome	CTP class A	CTP class B	CTP class C	Total
Death	00 (0.00%)	07 (18.92%)	17 (58.62%)	24 (16.00%)
Survival	77 (100.00%)	37 (81.08%)	12 (41.38%)	126 (84.00%)
Total	77 (100.00%)	44 (100.00%)	29 (100.00%)	150 (100%)

## Discussion

This was a retrospective study conducted on 150 CLD patients visiting a tertiary care centre over a period of two years. The most common age group was 41-60 years (86, 57.33%), followed by 21-40 (43, 28.67%) and 61-80 years (11, 07.33%). The mean age ± SD for all patients was 49.82 ± 11.63 years. In a study of 50 patients by Sreenivas et al., the mean age of presentation was 43.84 years with an SD of 10.4 years [6]. In a study by Cai et al., the mean age for liver cirrhosis was 48.27 ± 11.90 years and for acute on chronic liver disease, it was 51.14 ± 11.77 years [7]. Also, Sungkar et al. enrolled 54 liver cirrhosis patients and found the mean age for all patients was 52.76 ± 12.57 years [8]. Acharya et al. showed that out of 171 patients, the age range was 20-80 years and their mean age ± SD was 48.94 ± 12.63 years (median age = 50 years; range = 20-80 years) [9]. Thus, the mean age of presentation in our study was like the other studies.

In the present study, most CLD patients were males (96, 64%). Similar results were seen in a study by Sreenivas et al. who included 50 patients, out of which 46 (92%) were males and four (8%) were females [6]. Also, Cai et al.'s study found that liver cirrhosis cases in males were 73.41% [7]. Out of the 54 patients with cirrhosis enrolled in a study by Sungkar et al., 37 were males (68.52%) and 17 were females (31.48%) [8]. Arul et al.'s study included 100 patients of CLD, out of which 15% were females and 85% were males [10] and Zakareya et al. showed that out of 200 patients, males were 120 (60%) and females were 80 (40%) [11]. Thus, the male preponderance of cases of CLD in our study matches those of other studies and this was probably due to higher alcohol-related CLD in males.

In the present study, the most common cause of CLD was alcohol (76, 50.67%), followed by NAFLD (37, 24.67%). Hepatitis B, hepatitis C, cryptogenic, and autoimmune CLD cases were 11 (07.33%), seven (04.67%), 15 (10.00%), and four (02.66%), respectively. Sreenivas et al. showed that out of 50 CLD cases, alcohol was the aetiological factor in 41 patients, HCV infection in three patients, HBV infection in one patient, alcohol and HBV in three patients, and alcohol and HCV infection in one patient, respectively [6]. In contrast to the present study, Sungkar et al. enrolled 54 patients with cirrhosis and found that HBV infection (53.7%) was the major infection followed by an unknown causative agent (non-B and C) in 44.4% of patients [8]. Also, Acharya et al. showed that among 171 patients, end-stage liver disease was caused by alcohol in 88 (51.46%) patients and by both alcohol and a virus in 11 (6.43%) patients. A pure viral aetiology was present in 51 patients (29.82%), i.e., hepatitis B in 36 cases (21.05%), hepatitis C in 11 cases (6.43%), hepatitis B + C in three cases (1.75%), and hepatitis B + A in one case (0.58%) [9]. End-stage liver disease was due to non-alcoholic steatohepatitis in 13 (7.60%) patients, autoimmune hepatitis in seven (4.09%) patients, and Wilson’s disease in one (0.58%) patient [9].

In the present study, most CLD patients presented with generalized weakness (144, 96.00%). In contrast to the present study, Sreenivas et al., who included 50 patients with CLD, showed that the most common clinical presentation was abdominal distension (94%), followed by jaundice (56%) [6]. Acharya et al. showed that the most common finding was jaundice (69.00%), followed by ascites (63.15%), hepatic encephalopathy (32.74%), melena (29.82%), and hematemesis (20.46%) [9]. The present study showed that the most common clinical signs were icterus (68, 45.33%) and ascites (44, 29.33%). In contrast, Acharya et al. showed that the most common signs seen among the patients with end-stage liver disease were icterus (69.00%), pallor (28.07%), splenomegaly (25.14%), and parotid enlargement (23.39%) [9].

UGI endoscopy findings in the present study showed overlapping findings, out of which the most common were portal hypertensive gastropathy (either mild or severe) (135, 90.00%), followed by grade I varices (63, 42.00%). In contrast to the present study, Sreenivas et al. found that out of 50 patients with CLD, most patients showed normal UGI endoscopy (18, 38%), followed by grade II varices (13, 28.5%) and grade IV varices (5, 11.60%), respectively [6].

The present study showed that the mean bilirubin in CTP classes A, B, and C was 02.14 ± 00.87, 02.67 ± 01.07, and 03.65 ± 01.57 md/dl, respectively; mean albumin was 03.07 ± 01.11, 02.32 ± 01.01, and 01.92 ± 00.72 gm/dl; mean ALT was 41.24 ± 10.54, 47.24 ± 09.82, and 44.11 ± 06.54 IU/L; mean AST was 47.81 ± 07.63, 58.47 ± 09.77, and 51.24 ± 10.24 IU/L; mean haemoglobin was 10.12 ± 02.12, 09.14 ± 02.54, and 08.41 ± 02.55 gm/dl; mean WBC was 07.55 ± 05.11, 08.41 ± 04.21, and 08.74 ± 03.87 x 103/ml; mean platelets level was 102.50 ± 24.12, 98.20 ± 31.24, and 78.24 ± 21.14; mean INR was 01.41 ± 00.41, 01.31 ± 00.57, and 01.87 ± 00.77; mean serum creatinine (mg/dl) was 01.21 ± 00.21, 01.31 ± 00.57, and 01.61 ± 00.65 x103/ml; mean Na was 128.54 ± 03.21, 126.41 ± 03.11, and 125.41 ± 02.41 mEq/l; and mean K was 04.41 ± 01.11, 04.25 ± 01.23, and 04.88 ± 01.57 mEq/l, respectively. The above findings were statistically significant when compared with healthy individuals with a p-value < 0.001, except for serum creatinine and serum potassium, which showed a p-value > 0.05. Similarly, Tawfik et al. [12] and Sreenivas et al. [6] found comparable results.

In the present study, the total number of deaths out of the 150 patients was 24 (16.00%). The total number of deaths among 29 patients in CTP class C was 17 (70.83% of the total deaths) and among 44 patients in CTP class B was seven (29.16%), respectively. No deaths were seen in CTP class A. Sreenivas et al. showed that the total number of deaths was 12 (24%) [6]. The number of deaths among 18 patients in class B was one (8.33% of the total deaths) and deaths among 32 patients in class C were 11 (91.66%). The number of deaths in CTP class B was compared with the number of deaths in CTP class C. Thus, increased deaths in CTP class C in our study matched with the study by Sreenivas et al. [6].

## Conclusions

The study observed 150 CLD patients visiting a tertiary care centre in eastern India over a two-year period. The most common age group was 41-60 years, and the majority of patients were male. Alcohol was the most common cause of CLD, followed by NAFLD. A significant increasing trend of ALD-related morbidity and mortality was observed in this study. The study provides demographics and clinical features of CLD patients in this group of population and underscores the need for preventive measures, early detection, and medical attention to address the burden of this condition.
